# Recent Advances in Stochastic Sensor Control for Multi-Object Tracking

**DOI:** 10.3390/s19173790

**Published:** 2019-09-01

**Authors:** Sabita Panicker, Amirali Khodadadian Gostar, Alireza Bab-Hadiashar, Reza Hoseinnezhad

**Affiliations:** School of Engineering, RMIT University, Victoria 3083, Australia

**Keywords:** stochastic sensor control, PHD filter, multi-Bernoulli filter, random finite sets, multi-target tracking

## Abstract

In many multi-object tracking applications, the sensor(s) may have controllable states. Examples include movable sensors in multi-target tracking applications in defence, and unmanned air vehicles (UAVs) as sensors in multi-object systems used in civil applications such as inspection and fault detection. Uncertainties in the number of objects (due to random appearances and disappearances) as well as false alarms and detection uncertainties collectively make the above problem a highly challenging stochastic sensor control problem. Numerous solutions have been proposed to tackle the problem of precise control of sensor(s) for multi-object detection and tracking, and, in this work, recent contributions towards the advancement in the domain are comprehensively reviewed. After an introduction, we provide an overview of the sensor control problem and present the key components of sensor control solutions in general. Then, we present a categorization of the existing methods and review those methods under each category. The categorization includes a new generation of solutions called selective sensor control that have been recently developed for applications where particular objects of interest need to be accurately detected and tracked by controllable sensors.

## 1. Introduction

The sensor control problem is often considered under the umbrella of “Sensor Management”, which often deals with a wide range of management issues related to sensors in applications such as sensor scheduling, communication protocols, and sensor kinematics. Sensor control in multi-object tracking systems, on the other hand, presents a very specific domain which pays exclusive attention to the applications that need sensor control implementation, where all deployed sensors are efficiently utilized to gain a comprehensive understanding of the tracking scenario.

The term ‘Sensor Control’, in the context of this paper, refers to the dynamic deployment of one or more sensors in various multi-object tracking scenarios, by seeking the best control command for achieving optimum tracking results. The control command refers to the control of the freedom of movement of mobile sensors in a sensing system, to efficiently track the objects within the operational constraints of the system. The sensor control strategies are devised using stochastic methods, given the highly probabilistic scenario posed by the unpredictability of the noise, clutter and the object behaviors.

This review focuses on stochastic control solutions for sensor movements in multi-object systems that are modeled in the Random Finite Set (RFS) framework. Such solutions are usually formulated based on the widely followed Partially Observable Markov Decision Process (POMDP) [1] approach for Bayesian multi-object filtering, in which each action is perceived as a result of the previous measurement.

Although the broader area of sensor management has seen a tremendous growth in the past decade and has been reviewed in several papers in the literature [2,3,4,5,6,7,8,9,10,11,12,13,14,15,16], there seems to be no comprehensive review of the specific area of stochastic sensor control that provides detailed insight into the contributions and progress made in this domain. As such, in this work, we present the general sensor control problem and the key components of typical solutions introduced in the literature. This is followed by a categorization of the existing methods that include a new generation of solutions called selective sensor control.

The broader area of sensor management has seen tremendous growth in the past few decades, and the solutions developed for conventional multi-object filters have been reviewed in several papers in the literature [1,2,3,5,17,18,19,20,21,22,23].  Therefore, although we consider those methods in our categorization, we do not include a a review of them in this article, and concentrate on the more recent literature that is focused on sensor control for RFS-based filters.

The paper is organized as follows. Section 2 presents an overview of the sensor control problem formulation and the key concepts. Section 3 provides a categorization of sensor control approaches. This is followed by Section 4 in which a review of the major contributions in each category is provided. Some comparative simulation experiments and their results are presented and discussed in Section 5, followed by concluding remarks and future directions presented in Section 7.

## 2. Sensor Control Problem

The multi-object sensor control is a nonlinear stochastic control problem that aims to assign sensors the right sensor state at the right time [3]. The control problem is particularly challenging due to the high complexity and uncertainties involved in multi-object systems. The uncertainty is introduced into the system by the unperceived variations in the number of objects and also the false alarms and misdetections inherent to the system dynamics.

The sensor control decision is made in the presence of uncertainties in the object state and measurement spaces, usually with the assumption that the previous observation is available when making the next decision. Such stochastic control problems can be effectively handled in the POMDP framework [1] where the multi-object state is modeled as a Markov process, with knowledge on the posterior probability density function (pdf) of the multi-object state conditioned on the past measurements and the true state being unknown. The solution to sensor control problems generally depends on the definition of a measure of goodness that is usually quantified in terms of either the accuracy of the resulting multi-object state estimation, or the information content of the resulting multi-object posterior distribution. The decision-making is performed based on optimizing an *objective function*.

**Figure 1:**
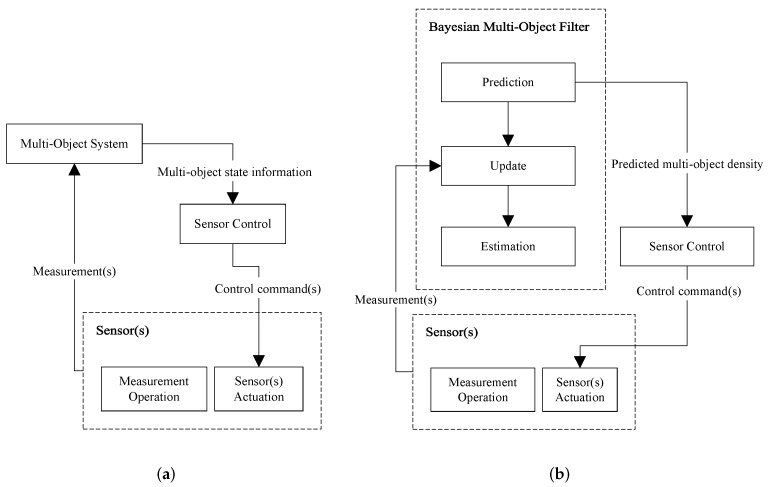
(**a**) general block diagram of a multi-object system with sensor control; (**b**) common data flow with a Bayesian multi-object filter used to implement the multi-object system.

Consider a general multi-object system. As shown in the schematic diagram of Figure 1a, it receives measurements from sensor(s) and outputs’ multi-object state information at any time. The sensor control solution inputs that information and outputs control command(s) for the sensor(s) that are then actuated accordingly, before the next measurements are acquired and sent to the multi-object system for processing at the next iteration.

**Figure 2:**
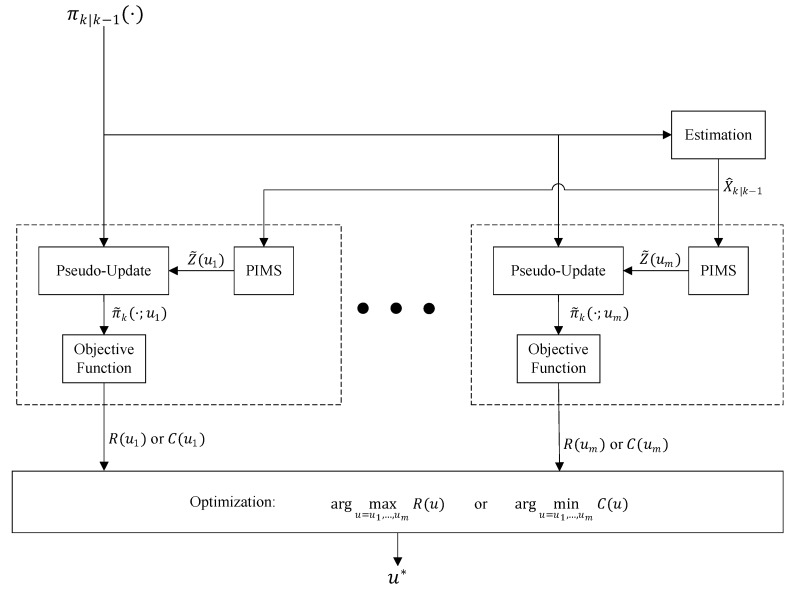
The most common approach to implement the “Sensor Control” block in Figure 1b.

In many applications, the multi-object system is implemented as a Bayesian multi-object filter that is mainly comprised of three steps: prediction, update, and estimation. In such scenarios, the sensor control problem turns into a stochastic control solution. Figure 1b shows a schematic diagram of the complete closed-loop system, exhibiting the usual approach in which the sensor control module constructs the control commands from the predicted multi-object density. Figure 2 presents how this information is processed inside a generic sensor control algorithm to generate a control command decision in the single-sensor case. Note that the outputs of the “estimation block” are only determined after the sensor control commands are determined and sensors are actuated accordingly then measurements are acquired using which the update step is executed. Importantly, the outputs of the “Estimation” block cannot be directly used for sensor control. However, the prediction step does not need the sensor measurements and can occur before any determination occurs on the sensor control commands. Hence, the most informative (and most recently updated) input that can be given to the sensor control block in Figure 1b is the “Predicted multi-object density”. Let us denote the predicted multi-object density at time *k* by $\pi_{k|k-1}\left(\cdot \right)$ and the set of all admissible sensor control commands by $U=\{u_{1},\ldots ,u_{m}\}$. The predicted density is used first to extract a predicted set of object state estimates, denoted by ${\hat{X}}_{k|k-1}$. For each possible control command $u_{i}\in U$, the following steps are taken:

First, a set of ideal noise-free and clutter-free measurements called the predicted ideal measurement set (PIMS) [6], denoted by $\tilde{Z}\left(u_{i}\right),$ is constructed as follows:

(1) $$\begin{array}{ccc} \tilde{Z}\left(u_{i}\right) & = & {\left\{\tilde{z}\left(\hat{x};u_{i}\right)\right\}}_{\hat{x}\in {\hat{X}}_{k|k-1}},\\ & & \mathrm{where}\\ \tilde{z}\left(\hat{x};u_{i}\right) & = & \underset{z}{arg max}g\left(z|\hat{x};u_{i}\right), \end{array}$$

in which $g(z|\hat{x};u_{i})$ is the single-object likelihood function (sensor model) which varies with the control command $u_{i}$.

**Example** **1.** *Consider a multi-object tracking application in 2D space, where each single-object state contains the object location coordinates (e.g., its x and y if the object is moving in a 2D space) and may include other components such as velocity, acceleration, and bearing angle. We also consider a sensor that its location is controllable, i.e., the control command $u_{i}$ is the sensor location vector. The sensor measures range. That is, if it detects the object, it returns:*

(2) z=ρ+n,

*where $\rho =\left|\right|\hat{x}-u_{i}{\left|\right|}_{2}$ and the measurement noise n is a zero-mean Gaussian random variable with a range dependent variance $\sigma {\left(\rho \right)}^{2}$. The single-object likelihood function is then given by:*

(3) $$g\left(z|\hat{x};u_{i}\right)=\frac{1}{\sqrt{2\pi }\;\sigma \left(\rho \right)}\exp\left(-\frac{{\left(z-\rho \right)}^{2}}{2\;\sigma {\left(\rho \right)}^{2}}\right).$$

*Hence, for a given sensor location $u_{i}$ and object location $\hat{x}$, the PIMS measurement is given by:*

(4) $$\tilde{z}\left(\hat{x};u_{i}\right)=\rho =\left|\right|\hat{x}-u_{i}{\left|\right|}_{2},$$

*which achieves the maximum likelihood value of*

(5) $$g_{\max}\left(\hat{x};u_{i}\right)=\frac{1}{\sqrt{2\pi }\sigma \left(\rho \right)}.$$

After the PIMS is calculated, the update step of the multi-object filter is run. Since this update step utilizes the PIMS as a measurement set, we call it *pseudo*-update and its output is called a *pseudo*-posterior denoted by ${\tilde{\pi }}_{k}\left(\cdot ;u_{i}\right)$ (as shown in Figure 2). An essential component of the algorithm is an objective function that inputs this pseudo-posterior and outputs a cost value, $C(u_{i})$, or a reward $R(u_{i})$. An optimization search over all the cost or rewards for various admissible control commands returns the *best* control command $u^{*}$. This command can be one of various actions such as spinning in positive or negative directions or making a step displacement in different directions.

## 3. Sensor Control Categorization

**Figure 3:**
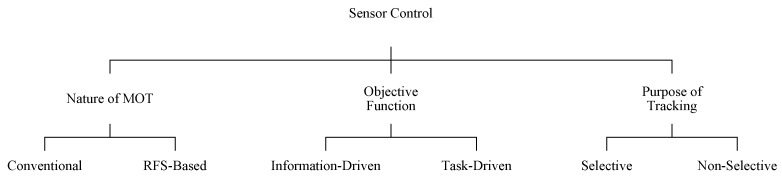
Classification of recent advanced sensor control solutions for multi-object tracking systems.

In this section, we present the sensor control methods that have been proposed in the recent literature, in three classes based on the following criteria: (a) *nature of multi-object tracking (MOT)*, (b) *objective function* and (c) *purpose of tracking*. As presented in Figure 3, in each class, there are a few sub-classes.

### 3.1. Nature of MOT

This review focuses on sensor control for applications where the multi-object system is a Bayesian multi-object filter—see Figure 1b. In such applications, the filter is usually designed to track the objects/targets, i.e., not only to estimate the number and states of the objects, but also to give them labels at each time. Therefore, the multi-object system block in Figure 1a,b are indeed multi-object trackers (MOTs).

The design of the sensor control method is heavily dependent on the nature of the MOT used in the closed-loop control system. In this study, we classify the sensor control methods into solutions that work with: (a) *conventional* and (b) *Random Finite Set* (RFS)-based multi-object trackers (also called multi-target filters in some papers).

The conventional multi-target filtering and sensor control methods use approaches such as Global Nearest Neighbor (GNN), Joint Probabilistic Data Association (JPDA) filter, and Multiple Hypothesis Tracking (MHT) [24] for finding associations between targets and measurements. In these methods, the uncertainty caused by difficulties in data association pose a major challenge along with the exponential growth in computations related to the number of targets and measurements.

As multi-target tracking deals with highly complex multi-object stochastic systems in which the number of objects vary randomly in time, and the measurements are subject to missed detections and false alarms, it would be best handled with a unified framework that extends the single-target tracking approach to multiple targets. This can be achieved by representing the multi-target states and observations as a random finite set (RFS) [3] in the finite set statistics (FISST) framework [25]. The RFS-based approach provides a principled and rigorous framework for modeling the multi-object systems and devising efficient control methods.

### 3.2. Objective Function

The essential part of any sensor control solution is its decision-making component, which selects the optimal sensor command(s). Commonly, the decision-making is performed based on optimizing an objective function. Based on the objective function employed, there are two classifications presented in the sensor control literature: *Task-driven* and *Information-driven* methods.

In the task-driven method, the objective function is formulated as a cost function that directly depends on the tracking performance quantified by metrics such as error variance and cardinality variance [16,26]. In the case of information-driven methods, the focus is on the information content of the multi-object posterior resulting from the Bayesian update with measurements obtained after sensor control. In this case, the objective function is a reward function, which is usually computed using a measure of information gain from prior to posterior. Some examples of reward functions are Shannon entropy [27], Kullback–Leibler divergence [2,28], Rényi divergence [11,12] and Cauchy–Schwarz divergence [29].

### 3.3. Purpose of Tracking

Apart from the two classifications discussed above, a new classification based on the *purpose of tracking* has come into vogue, which stems from the recently introduced *selective sensor control* methods [16]. This groups the sensor control methods into *selective* and *non-selective* types.

#### 3.3.1. Non-Selective Sensor Control

This is the conventional sensor control method which aims at efficiently tracking a group of targets by positioning the sensors towards the direction of targets, in the case of non-mobile sensors; and towards the center of target locations, in case of mobile sensors. Several works exist in the literature that have enriched the domain of *non-selective sensor control* [1,2,3,5,6,11,26,28,30,31,32,33,34,35,36].

#### 3.3.2. Selective Sensor Control

While the conventional sensor control strategies are designed to manage sensors in tracking a set of targets, *selective sensor control methods* focus on tracking scenarios where only a specific target or a selected subset of targets in the surveillance area is of primary interest. In such applications, the goodness of sensor measurements is of primary concern for tracking only the specific targets of interest (ToIs), and sensor control is performed to obtain better measurements for the ToIs. The availability of labeled random finite set filters [37,38,39,40,41] have made it possible to track the trajectories of targets apart from estimating their number and states [37,38,40,41,42]. For instance, the target label information returned by a Labeled Multi-Bernoulli (LMB) filter can be effectively used for sensor control in selective target tracking applications.

## 4. Survey of Recent Literature

In this section, we explore some of the significant methods that have been recently proposed in the signal processing literature for single and multi-sensor control for multi-target tracking in a stochastic POMDP framework. Table 1 lists how each of the methods that are reviewed in this article, are classified. In all these methods, the sensor control solution is primarily proposed to be used with RFS-based multi-target tracking filters. However, Mahler’s method called Posterior Expected Number of Targets (PENT) [5] can be simply used with both the RFS-based multi-target filters (such as the probability hypothesis density—PHD—filter) and the conventional ones (such as the MHT filter). This section would provide the reader with a snapshot of the significant steps in the evolution of sensor control methods to the current state-of-the-art.

### 4.1. Information-Driven Sensor Control

The purpose of sensor control is to effectively use the sensor/s, by allowing them to interact with the tracking environment to gain useful information that reduce uncertainty. Every movement of the sensor is aimed at an information gain on the targets’ states and cardinality i.e., information on the accuracy of state/location of the target that is being tracked; and/or information on the presence or absence of targets. The amount of information gain is usually measured by the change in entropy between the probability densities prior and post sensor measurements. It was in the work of Hintz and McVey [46] that we find the earliest reference to the application of information-driven control for sensor management and state estimation, or, more precisely, the measures for information gain, where they employed Shannon entropy with Kalman filters for target tracking.

A number of information theoretic divergence measures followed that, for sensor control in single and multi-target tracking scenarios. These included divergence methods like the Kullback–Leibler (KL) divergence [17] and Rényi divergence [47] between the prior and posterior target densities. The expected divergence or information gain (that measures the difference between information content of random variables) is thus used as the basis for selecting the optimal control action. Similarity between random variables can also be measured using the distance between their probability distributions, as in methods like total variation, Bhattacharyya, Hellinger-Matusita, Wasserstein, etc. These measures cannot be computed analytically and hence require expensive approximations like Monte Carlo, except for some special cases. In this section, we endeavor to briefly touch upon information-driven sensor control methods due to their popular usage.

#### 4.1.1. Rényi Divergence-Based Sensor Control

The Rényi or alpha divergence is a commonly used objective function in information-driven sensor control. It is defined as a measure of information gain from a prior to a posterior multi-target density. Referring to Figure 2, let us assume that, at time *k*, for a given sensor control command, *u*, the predicted multi-object density $\pi_{k|k-1}\left(\cdot \right)$ evolves to a pseudo-posterior ${\tilde{\pi }}_{k}\left(\cdot ;u\right)$ after going through a pseudo-update step using the PIMS $\tilde{Z}\left(u\right)$. The information gain from the prior to the pseudo posterior (which is the information gained from PIMS measurements) is quantified by the Rényi divergence between the two densities, defined as follows:

(6) $$R\left(u\right)=\frac{1}{\alpha -1}\log\left(\int [{\tilde{\pi }}_{k}{\left(X;u\right)]}^{\alpha }{\left[\pi_{k|k-1}\left(X\right)\right]}^{1-\alpha }\delta X\right),$$

where α∈(0,1) is an adjustable parameter. The Rényi divergence turns into the Kullback–Leibler divergence when α→1 or the Hellinger affinity with α=0.5. Set integration is generally defined as follows: [42]

(7) $$\int_{S}f\left(X\right)\delta X=f\left(\emptyset \right)+\int_{S}f\left(x\right)dx+\frac{1}{2!}\int_{S\times S}f\left(x_{1},x_{2}\right)dx_{1}dx_{2}+\frac{1}{3!}\int_{S\times S\times S}f\left(x_{1},x_{2},x_{3}\right)dx_{1}dx_{2}dx_{3}+\cdots .$$

#### Rényi Divergence-Based Sensor Control with a General RFS Filter

In 2010, Ristic and Vo [11] proposed an information-driven sensor control method to propagate the multi-object posterior of the particle implementation of Bayes multi-object filter using random finite sets. They employed Rényi divergence as reward function in their implementation. As there is no general closed-from solution to Rényi divergence, a numerical approximation using a Sequential Monte Carlo (SMC) method is provided in the paper. Assume that the prior distribution is approximated by *N* particles:

(8) $$\pi_{k|k-1}\left(X\right)≊\sum_{i=1}^{N}w_{i}\delta \left(X-X_{i}\right),$$

where each pair $\left(X_{i},w_{i}\right)$ represents a multi-object set particle and its weight. Through application of Bayes’ rule (which is related the posterior to the prior through the multi-object likelihood function), the Rényi divergence is proven to be approximated by:

(9) $$R\left(u\right)≊\frac{1}{\alpha -1}\log\frac{\sum_{i=1}^{N}w_{i}{\left[g\left(\tilde{Z}\left(u\right)|X_{i},u\right)\right]}^{\alpha }}{{\left[\sum_{i=1}^{N}w_{i}g\left(\tilde{Z}\left(u\right)|X_{i},u\right)\right]}^{\alpha }},$$

where g(Z|X,u) is the multi-object likelihood function (sensor model). The optimal control vector is chosen by:

(10) $$u^{*}=\underset{u\in U}{arg max}\left[R(u)\right].$$

#### Rényi Divergence-Based Sensor Control with a PHD/CPHD Filter

Approximating a multi-object density by particle sets is evidently very expensive in terms of computation. Indeed, the original method proposed in [11] involved sampling in the measurement set as well, which can be simplified using the PIMS instead. However, the computational cost can be too heavy to implement in the presence of more than five objects in the scene. Later in 2011, Ristic and Vo [12] proposed the Poisson and i.i.d. cluster approximation of Rényi divergence for sensor control with multi-object tracking using PHD and Cardinalized PHD (CPHD) filters.

Assume that both the predicted and updated multi-object densities are of i.i.d. cluster types given by:

(11) $$\pi_{k|k-1}\left(X\right)=n!\rho_{0}\left(n\right)\prod_{x\in X}s_{0}\left(x\right),$$

(12) $${\tilde{\pi }}_{k}\left(X;u\right)=n!\rho_{1}\left(n;u\right)\prod_{x\in X}s_{1}\left(x;u\right),$$

where ρ(n) and s(x) are the cardinality distribution and the spatial single-object density, respectively. The Rényi divergence for i.i.d. cluster pdfs applicable to the CPHD filter recursions is derived as [12]

(13) $$R\left(u\right)=\frac{1}{\alpha -1}\log\sum_{n=0}^{\infty }\rho_{1}{\left(n;u\right)}^{\alpha }\rho_{0}{\left(n\right)}^{1-\alpha }{\left[\int s_{1}{\left(x;u\right)}^{\alpha }s_{0}{\left(x\right)}^{1-\alpha }dx\right]}^{n}.$$

In PHD filter recursion where both the multi-object densities are Poisson RFS densities, the Rényi divergence becomes simpler as follows:

(14) $$R\left(u\right)=\lambda_{0}+\frac{\alpha }{1-\alpha }\lambda_{1}\left(u\right)+\frac{\lambda_{1}{\left(u\right)}^{\alpha }\lambda_{0}^{1-\alpha }}{\alpha -1}\int s_{1}{\left(x;u\right)}^{\alpha }s_{0}{\left(x\right)}^{1-\alpha }dx,$$

where λ is the average number of objects in the Poisson RFS and s(x) is the spatial single-object density. In their works, Ristic and Vo [11,12] examine the reward function in a scenario involving multiple moving objects and a controllable moving range-only sensor. The sensor’s detection accuracy (misdetection, noise and false alarm rate) improves for shorter sensor-object distance. Hence, in this case, the sensor control algorithm is expected to drive the sensor as close as possible to all objects. The Rényi divergence in this case is seen to be rapidly controlling the sensor to move towards the objects. Consequently, the optimal sub-pattern assignment (OSPA) metric, which is used as for performance evaluation, decreases when the sensor control algorithm is in place.

Though several solutions for sensor control using Rényi divergence have been proposed, the obvious problem with all these is the computational expense associated with them, primarily due to the absence of a robust analytical solution to Rényi divergence.

#### 4.1.2. Cauchy–Schwarz Divergence

Another information theoretic reward function that has attracted attention recently is the Cauchy–Schwarz (CS) divergence. The CS divergence is based on the Cauchy–Schwarz inequality for inner products, and, for two random vectors with probability densities *f* and *g*, it is defined as [29]

(15) $$D_{CS}\left(f,g\right)=-\ln\frac{\langle f,g\rangle }{∥f∥∥g∥},$$

where $\langle f,g\rangle ≜\int f\left(x\right)g\left(x\right)dx$ is the inner product of the two functions, and $\left||f|\right|≜\sqrt{\langle f,f\rangle }$. The CS divergence is a measure of the distance between the two densities. It is to be noted that, when f=g, $D_{CS}\left(f,g\right)=0$; otherwise, it is positive and a symmetric function. The argument of the logarithm in Equation (15) does not exceed one (according to Cauchy–Schwarz inequality) and is positive, as probability densities are non-negative. Geometrically, in CS divergence, the logarithm argument is the cosine of the angle between the two densities in the space of density functions, hence representing the “difference” in information content of the two densities. The Cauchy–Schwarz divergence can also be interpreted as an approximation to the Kullback–Leibler divergence [17].

#### CS Divergence-Based Sensor Control with a PHD Filter

Consider the predicted and pseudo-posterior multi-object RFS densities, $\pi_{k|k-1}\left(\cdot \right)$ and ${\tilde{\pi }}_{k}\left(\cdot ;u\right)$. Hoang et al. [31] showed that the CS divergence between the two RFS densities is given by:

(16) $$D_{CS}\left(\pi_{k|k-1}\left(\cdot \right),{\tilde{\pi }}_{k}\left(\cdot ;u\right)\right)=-\ln\frac{\int K^{\left|X\right|}\pi_{k|k-1}\left(X\right){\tilde{\pi }}_{k}\left(X;u\right)\delta X}{\sqrt{\left[\int K^{\left|X\right|}{\left(\pi_{k|k-1}\left(X\right)\right)}^{2}\delta X\right]\left[\int K^{\left|X\right|}{\left({\tilde{\pi }}_{k}\left(X;u\right)\right)}^{2}\delta X\right]}},$$

where *K* is the unit of hyper-volume in the single-object state space.

Hoang et al. [31] derived a closed-form solution for the CS divergence between two Poisson RFS densities and proposed how it could be used for sensor control with the PHD filter [4,7]. Assume that the predicted prior and pseudo posterior densities are Poisson densities in a PHD filter, with their intensity functions denoted by $v_{k|k-1}\left(x\right)$ and $\tilde{v_{k}}\left(x;u\right)$. Note that with the intensity function v(·) given, the average cardinality λ and spatial single-object density s(·) can be computed as follows:

(17) $$\lambda =\int v\left(x\right)dx;\;\;s\left(x\right)=\frac{v(x)}{\int v(y)dy}.$$

Honag et al. [31] proved that the CS divergence between the two Poisson RFS densities is simply given by:

(18) $$\begin{array}{ccc} R\left(u\right)=D_{\mathrm{CS}}\left(\pi_{k|k-1}\left(\cdot \right),{\tilde{\pi }}_{k}\left(\cdot ;u\right)\right) & = & \frac{K}{2}{∥v_{k|k-1}\left(\cdot \right)-{\tilde{v}}_{k}\left(\cdot ;u\right)∥}^{2}\\ & = & \frac{K}{2}\int {\left|v_{k|k-1}\left(x\right)-{\tilde{v}}_{k}\left(x;u\right)\right|}^{2}dx, \end{array}$$

where *K* is the unit of hyper-volume measurement in the single-object state space. In simple terms, the Cauchy–Schwarz divergence between any two Poisson point processes is half the squared distance between their intensity functions. Sensor control takes place by choosing the control command that maximizes the above divergence as the reward function:

(19) $$u^{*}=\underset{u\in U}{arg max}\int {\left|v_{k|k-1}\left(x\right)-{\tilde{v}}_{k}\left(x;u\right)\right|}^{2}dx.$$

Calculation of the reward function depends on how the PHD filter is implemented. In an SMC implementation, suppose that the two intensity functions are approximated by the same particles but different weights. Note that this assumption is based on what actually happens in the filter because, through the update step, only particle weights are changed, not the location of the particles. Then we can assume:

(20) $$\begin{array}{ccc} v_{k|k-1}\left(x\right) & ≊ & \sum_{i=1}^{N}w_{k|k-1,i}\;\delta \left(x-x_{i}\right), \end{array}$$

(21) $$\begin{array}{ccc} \tilde{v_{k}}\left(x;u\right) & ≊ & \sum_{i=1}^{N}{\tilde{w}}_{k,i}\left(u\right)\;\delta \left(x-x_{i}\right). \end{array}$$

Then, the sensor control policy (19) is implemented as below:

(22) $$u^{*}=\underset{u\in U}{arg max}\sum_{i=1}^{N}{\left|w_{k|k-1,i}-{\tilde{w}}_{k,i}\left(u\right)\right|}^{2}.$$

For applications that require target tracks, this approach does not seem to fit, as the PHD filters do not provide tracks. In addition, the drawback with the PHD filter for sensor control is that it involves a poor approximation to the multi-target posterior, leading to highly uncertain cardinality estimates [8,9].

#### CS Divergence-Based Sensor Control with a Multi-Bernoulli Filter

Later in 2016, based on (18), Gostar et al. [35] derived an approximation for the CS divergence between two multi-Bernoulli densities and used it for sensor control in a cardinality-balanced multi-Bernoulli filter (CB-MeMBer). Consider the multi-Bernoulli (MB) predicted multi-object prior and pseudo posterior being parameterized as

(23) $$\begin{array}{ccc} \pi_{k|k-1}\left(\cdot \right) & = & {\left\{\left(r_{k|k-1}^{\left(i\right)},p_{k|k-1}^{\left(i\right)}\left(\cdot \right)\right)\right\}}_{i=1}^{M}, \end{array}$$

(24) $$\begin{array}{ccc} {\tilde{\pi }}_{k}\left(\cdot ;u\right) & = & {\left\{\left({\tilde{r}}_{k}^{\left(i\right)}\left(u\right),{\tilde{p}}_{k}^{\left(i\right)}\left(\cdot ;u\right)\right)\right\}}_{i=1}^{M}, \end{array}$$

where each $\left(r^{\left(i\right)},p^{\left(i\right)}\left(\cdot \right)\right)$ denotes a possible object (a Bernoulli component) with its probability of existence being $r^{\left(i\right)}$, and its state density function conditioned on existence being $p^{\left(i\right)}\left(\cdot \right)$. Gostar et al. [35] approximate each MB density with its closest Poisson RFS density, which is the Poisson with matching intensity function. The intensity function of an RFS density is its first moment, and, for the above two MB densities, they are given by:

(25) $$\begin{array}{ccc} v_{k|k-1}\left(x\right) & = & \sum_{i=1}^{M}\left(r_{k|k-1}^{\left(i\right)}\;p_{k|k-1}^{\left(i\right)}\left(x\right)\right), \end{array}$$

(26) $$\begin{array}{ccc} {\tilde{v}}_{k}\left(x;u\right) & = & \sum_{i=1}^{M}\left({\tilde{r}}_{k}^{\left(i\right)}\left(u\right)\;{\tilde{p}}_{k}^{\left(i\right)}\left(x;u\right)\right). \end{array}$$

Substituting the intensity functions in Equation (22) results in the following sensor control policy:

(27) $$u^{*}=\underset{u\in U}{arg max}\int {\left|\sum_{i=1}^{M}\left(r_{k|k-1}^{\left(i\right)}\;p_{k|k-1}^{\left(i\right)}\left(x\right)-{\tilde{r}}_{k}^{\left(i\right)}\left(u\right)\;{\tilde{p}}_{k}^{\left(i\right)}\left(x;u\right)\right)\right|}^{2}dx.$$

Again, to calculate the integral, in an SMC implementation, suppose that single-object density of each Bernoulli component is approximated by particles:

(28) $$\begin{array}{ccc} p_{k|k-1}^{\left(i\right)}\left(x\right) & ≊ & \sum_{j=1}^{J^{\left(i\right)}}w_{k|k-1,j}^{\left(i\right)}\;\delta \left(x-x_{j}^{\left(i\right)}\right), \end{array}$$

(29) $$\begin{array}{ccc} {\tilde{p}}_{k}^{\left(i\right)}\left(x;u\right) & ≊ & \sum_{j=1}^{J^{\left(i\right)}}{\tilde{w}}_{k,j}^{\left(i\right)}\left(u\right)\;\delta \left(x-x_{j}^{\left(i\right)}\right). \end{array}$$

Then, the sensor control policy (27) is implemented as below:

(30) $$u^{*}=\underset{u\in U}{arg max}\sum_{i=1}^{M}\;\sum_{j=1}^{J^{\left(i\right)}}{\left(r_{k|k-1}^{\left(i\right)}\;w_{k|k-1,j}^{\left(i\right)}-{\tilde{r}}_{k}^{\left(i\right)}\left(u\right)\;{\tilde{w}}_{k,j}^{\left(i\right)}\left(u\right)\right)}^{2}.$$

It is important to note that, through the process of computing the PIMS and running the pseudo-update step, each Bernoulli component is assigned a measurement, i.e., data association is known. Hence, for each particle weight $w_{k|k-1,j}^{\left(i\right)}$, finding its associated weight in the updated MB, ${\tilde{w}}_{k,j}^{\left(i\right)}\left(u\right)$, is straightforward. See [35] for more details.

#### CS Divergence-Based Sensor Control with a Labeled Multi-Bernoulli Filter

The above investigation was followed by another approximate solution for the CS divergence between Poisson approximations of the predicted and updated Sequential Monte Carlo (SMC) Labeled Multi-Bernoulli (LMB) posteriors [45]. Consider the LMB predicted multi-object prior and pseudo posterior being parameterized as

$$\pi_{k|k-1}\left(\cdot \right)∼{\left\{\left(r_{k|k-1}^{\left(\ell \right)},p_{k|k-1}^{\left(\ell \right)}\left(\cdot \right)\right)\right\}}_{\ell \in L},\;\;\;{\tilde{\pi }}_{k}\left(\cdot ;u\right)∼{\left\{\left({\tilde{r}}_{k}^{\left(\ell \right)}\left(u\right),{\tilde{p}}_{k}^{\left(\ell \right)}\left(\cdot ;u\right)\right)\right\}}_{\ell \in L},$$

where each $\left(r^{\left(\ell \right)},p^{\left(\ell \right)}\left(\cdot \right)\right)$ denotes a possible target track (with label *ℓ*) with its probability of existence denoted by $r^{\left(\ell \right)}$, and its state density function conditioned on existence denoted by $p^{\left(\ell \right)}\left(\cdot \right)$. Note the change of density symbols to boldface, which is customary in the literature when denoting *labeled* densities. Similarly, labeled single-object and multi-object states are denoted by bold-face symbols x and X, respectively.

In a similar fashion to their previous work [35], Gostar et al. [45] showed that, with the above assumptions, the sensor control policy is derived as follows:

(31) $$u^{*}=\underset{u\in U}{arg max}\sum_{\ell \in L}\;\sum_{j=1}^{J^{\left(\ell \right)}}{\left(r_{k|k-1}^{\left(\ell \right)}\;w_{k|k-1,j}^{\left(\ell \right)}-{\tilde{r}}_{k}^{\left(\ell \right)}\left(u\right)\;{\tilde{w}}_{k,j}^{\left(\ell \right)}\left(u\right)\right)}^{2},$$

where $w_{k|k-1,j}^{\left(\ell \right)}$ and ${\tilde{w}}_{k,j}^{\left(\ell \right)}\left(u\right)$ are the weights of *j*-th particle which approximate the densities $p_{k|k-1}^{\left(\ell \right)}\left(\cdot \right)$ and ${\tilde{p}}_{k}^{\left(\ell \right)}\left(\cdot ;u\right)$ in an SMC implementation of the LMB filter, respectively.

#### CS Divergence-Based Sensor Control with a Generalized Labeled Multi-Bernoulli Filter

Beard et al. [33] derived the exact closed-form solution for CS divergence between two generalized labeled multi-Bernoulli (GLMB) densities for sensor control in applications where a GLMB filter [37,41], also known as the Vo–Vo filter is being used to track multiple targets. Let x=(x,ℓ) be a labeled single-object state and L(x)≜ℓ be a projection from the labeled state space to the label space, a function that takes the labeled state and returns the label part only. Denoting a labeled set by X, its label set is similarly defined as L(X)={L(x):x∈X}. For a labeled RFS, each element must have a distinct label, i.e., the cardinality of the set itself must equal the cardinality of its label set:

(32) $$\Delta \left(X\right)≜\left\{\begin{array}{cc} 1, & \mathrm{if}\;|X|=|L(X)|,\\ 0, & \mathrm{otherwise}. \end{array}\right.$$

The function Δ(X) is called the *distinct label indicator*. A GLMB is a labeled RFS with a density of the form:

(33) $$\pi \left(X\right)=\Delta \left(X\right)\sum_{c\in C}w^{\left(c\right)}\left(L\left(X\right)\right)\prod_{\left(x,\ell \right)\in X}p^{\left(c\right)}\left(x,\ell \right),$$

where C denotes a discrete and finite index set, each $p^{\left(c\right)}\left(x,\ell \right)$ is a density in the single-object state space, i.e., $\int p^{\left(c\right)}\left(x,\ell \right)dx=1$, and the label set weights $w^{\left(c\right)}\left(\cdot \right)$ are normalized over the space of labels and indexes, i.e.,

$$\sum_{c\in C}\sum_{L\in L}w^{\left(c\right)}\left(L\right)=1.$$

The above GLMB density is completely characterized by the set: $\left\{\left(w^{\left(c\right)}\left(L\right),p^{\left(c\right)}\left(\cdot \right)\right):c\in C,L\subseteq L\right\}.$

For detailed description of prediction and update steps of the GLMB filter and its particular form called delta-GLMB, refer to [37,41].

Assume that the predicted GLMB prior is characterized as:

$$\pi_{k|k-1}\left(\cdot \right)∼\left\{\left(w_{k|k-1}^{\left(c\right)}\left(L\right),p_{k|k-1}^{\left(c\right)}\left(\cdot \right)\right):c\in C_{k|k-1},L\subseteq L\right\}$$

and the pseudo-posterior associated with control command *u* is a GLMB characterized by:

$${\tilde{\pi }}_{k}\left(\cdot ;u\right)∼\left\{\left({\tilde{w}}_{k}^{\left(c^{\prime }\right)}\left(L;u\right),{\tilde{p}}_{k}^{\left(c^{\prime }\right)}\left(\cdot ;u\right)\right):c^{\prime }\in C_{k},L\subseteq L\right\}.$$

Beard et al. [33] showed that the CS divergence between the above GLMB densities is given by:

(34) $$R\left(u\right)=D_{\mathrm{CS}}\left(\pi_{k|k-1}\left(\cdot \right),{\tilde{\pi }}_{k}\left(\cdot ;u\right)\right)=-\ln\frac{{\langle \pi_{k|k-1}\left(\cdot \right),{\tilde{\pi }}_{k}\left(\cdot ;u\right)\rangle }_{K}}{\sqrt{{\langle \pi_{k|k-1}\left(\cdot \right),\pi_{k|k-1}\left(\cdot \right)\rangle }_{K}\;\;{\langle {\tilde{\pi }}_{k}\left(\cdot ;u\right),{\tilde{\pi }}_{k}\left(\cdot ;u\right)\rangle }_{K}}},$$

where

$$\begin{array}{ccc} {\langle \pi_{k|k-1}\left(\cdot \right),{\tilde{\pi }}_{k}\left(\cdot ;u\right)\rangle }_{K} & = & \sum_{L\subseteq L}\left[\sum_{\begin{array}{c} c\in C_{k|k-1}\\ c^{\prime }\in C_{k} \end{array}}\left(w_{k|k-1}^{\left(c\right)}\left(L\right)\;{\tilde{w}}_{k}^{\left(c^{\prime }\right)}\left(L;u\right)\;\prod_{\ell \in L}\langle p_{k|k-1}^{\left(c\right)}\left(\cdot ,\ell \right),{\tilde{p}}_{k}^{\left(c^{\prime }\right)}\left(\cdot ,\ell ;u\right)\rangle \right)\right],\\ {\langle \pi_{k|k-1}\left(\cdot \right),\pi_{k|k-1}\left(\cdot \right)\rangle }_{K} & = & \sum_{L\subseteq L}\left[\sum_{\begin{array}{c} c\in C_{k|k-1}\\ c^{\prime }\in C_{k|k-1} \end{array}}\left(w_{k|k-1}^{\left(c\right)}\left(L\right)\;w_{k|k-1}^{\left(c^{\prime }\right)}\left(L\right)\;\prod_{\ell \in L}\langle p_{k|k-1}^{\left(c\right)}\left(\cdot ,\ell \right),p_{k|k-1}^{\left(c^{\prime }\right)}\left(\cdot ,\ell \right)\rangle \right)\right],\\ {\langle {\tilde{\pi }}_{k}\left(\cdot ;u\right),{\tilde{\pi }}_{k}\left(\cdot ;u\right)\rangle }_{K} & = & \sum_{L\subseteq L}\left[\sum_{\begin{array}{c} c\in C_{k}\\ c^{\prime }\in C_{k} \end{array}}\left({\tilde{w}}_{k}^{\left(c\right)}\left(L;u\right){\tilde{w}}_{k}^{\left(c^{\prime }\right)}\left(L;u\right)\;\prod_{\ell \in L}\langle {\tilde{p}}_{k}^{\left(c\right)}\left(\cdot ,\ell ;u\right),{\tilde{p}}_{k}^{\left(c^{\prime }\right)}\left(\cdot ,\ell ;u\right)\rangle \right)\right]. \end{array}$$

SMC implementation of the above is straightforward. If each density is approximated with the same particles but different weights, each inner product of the densities is calculated by summing all the products of corresponding weights in the two sets of particles.

#### CS Divergence-Based Sensor Control with Constraints

An important consideration that has been taken into account by Beard et al. [33] and Gostar et al. [45] is the practical constraints that may be applicable when choosing the optimal sensor control command $u^{*}$. Imagine an application in which there is a region in the state space that the sensor cannot be controlled to end up there. For instance, in a defense application, if the objects are enemy targets, the sensors cannot end up up close to any of the targets. For each control command *u*, such a region can be denoted by ϵ(u). In such a constrained sensor control problem, we want the region to be void (empty of any objects) with a very high probability called the “void probability”. For a given multi-object density π(·), the void probability for a region *S* is defined as

(35) $$\psi_{\pi (\cdot )}\left(S\right)≜\int_{X-S}\pi \left(X\right)\delta X.$$

For the purpose of sensor control, the void probability is computed with the pseudo-posterior density in mind. The work in [45] suggests that, in multi-target tracking with LMB filters, implementation of sensor control with void probability constraint enables the control of sensors to be moved in desirable directions for maximizing the information gain, while keeping a safe distance from the targets. When the pseudo-posterior is of LMB form with parameters ${\left\{\left({\tilde{r}}_{k}^{\left(\ell \right)}\left(u\right),{\tilde{p}}_{k}^{\left(\ell \right)}\left(\cdot ;u\right)\right)\right\}}_{\ell \in L}$, and each density ${\tilde{p}}_{k}^{\left(\ell \right)}\left(\cdot ;u\right)$ is approximated with particles and weights ${\left\{\left(x_{k,j}^{\left(\ell \right)},{\tilde{w}}_{k,j}^{\left(\ell \right)}\left(u\right)\right)\right\}}_{j=1}^{J^{\left(\ell \right)}}$, the void probability is derived as follows [45]:

(36) $$\psi_{{\tilde{\pi }}_{k}\left(\cdot ;u\right)}\left(\epsilon (u)\right)=\prod_{\ell \in L}\left[1-{\tilde{r}}_{k}^{\left(\ell \right)}\left(u\right)\sum_{j=1}^{J^{\left(\ell \right)}}1_{\epsilon (u)}\left(x_{k,j}^{\left(\ell \right)}\right){\tilde{w}}_{k,j}^{\left(\ell \right)}\left(u\right)\right],$$

where

(37) $$1_{S}\left(x\right)≜\left\{\begin{array}{cc} 1, & \mathrm{if}\;x\in S,\\ 0, & \mathrm{otherwise}. \end{array}\right.$$

Taking the void probability constraint into account, the sensor control policy is turned into:

(38) $$u^{*}=\underset{u\in U_{\mathrm{constr}.}}{arg max}\sum_{\ell \in L}\;\sum_{j=1}^{J^{\left(\ell \right)}}{\left(r_{k|k-1}^{\left(\ell \right)}\;w_{k|k-1,j}^{\left(\ell \right)}-{\tilde{r}}_{k}^{\left(\ell \right)}\left(u\right)\;{\tilde{w}}_{k,j}^{\left(\ell \right)}\left(u\right)\right)}^{2},$$

where

(39) $$U_{\mathrm{constr}.}≜\left\{u\in U:\psi_{{\tilde{\pi }}_{k}\left(\cdot ;u\right)}\left(\epsilon (u)\right)>\psi_{\min}\right\}$$

and $\psi_{\min}$ is the minimum void probability, a user-defined threshold that is very close to 1.

When a GLMB filter is being used for multi-object tracking, the void probability is formulated differently. Consider the pseudo-posterior associated with control command *u* in which GLMB is characterized by:

$${\tilde{\pi }}_{k}\left(\cdot ;u\right)∼\left\{\left({\tilde{w}}_{k}^{\left(c\right)}\left(L;u\right),{\tilde{p}}_{k}^{\left(c\right)}\left(\cdot ;u\right)\right):c\in C_{k},L\subseteq L\right\}.$$

Beard et al. [33] show that the void probability in this case is given by:

(40) $$\psi_{{\tilde{\pi }}_{k}\left(\cdot ;u\right)}\left(\epsilon (u)\right)=\sum_{L\subseteq L}\left[\sum_{c\in C_{k}}\left({\tilde{w}}_{k}^{\left(c\right)}\left(L;u\right)\;\prod_{\ell \in L}\langle 1-1_{\epsilon (u)}\left(\cdot \right),{\tilde{p}}_{k}^{\left(c\right)}\left(\cdot ,\ell ;u\right)\rangle \right)\right].$$

In case of SMC implementation where each density ${\tilde{p}}_{k}^{\left(c\right)}\left(\cdot ,\ell ;u\right)$ is approximated by particles and weights ${\left\{\left({\tilde{x}}_{j}^{\left(\ell )(c\right)},{\tilde{w}}_{j}^{\left(\ell )(c\right)}\right)\right\}}_{j=1}^{J^{\left(\ell )(c\right)}}$, the above expression is simplified to:

(41) $$\psi_{{\tilde{\pi }}_{k}\left(\cdot ;u\right)}\left(\epsilon (u)\right)=\sum_{L\subseteq L}\left[\sum_{c\in C_{k}}\left({\tilde{w}}_{k}^{\left(c\right)}\left(L;u\right)\;\prod_{\ell \in L}\sum_{j=1}^{J^{\left(\ell )(c\right)}}\left(1-1_{\epsilon (u)}\left({\tilde{x}}_{j}^{\left(\ell )(c\right)}\right)\right){\tilde{w}}_{j}^{\left(\ell )(c\right)}\right)\right].$$

In this case, the control policy is:

(42) $$u^{*}=\underset{u\in U_{\mathrm{constr}.}}{arg max}D_{\mathrm{CS}}\left(\pi_{k|k-1}\left(\cdot \right),{\tilde{\pi }}_{k}\left(\cdot ;u\right)\right),$$

where $D_{\mathrm{CS}}\left(\pi_{k|k-1}\left(\cdot \right),{\tilde{\pi }}_{k}\left(\cdot ;u\right)\right)$ is given by Equation (34) and $U_{\mathrm{constr}.}$ is the set of all control commands u∈U for which the void probability calculated by (41) is more than the user-defined minimum threshold $\psi_{\min}$.

### 4.2. Task-Driven Sensor Control

The RFS-based sensor control methods, in general and task-driven sensor control approaches specifically, saw their beginnings in the works published by Mahler in 2003 [3] and 2004 [5], which provided the early investigations to devise a foundational basis for sensor management based on the RFS filtering framework. In the task-driven approach, the objective function is formulated as a cost function that directly depends on the tracking performance of the system, quantified by metrics such as error and cardinality variance.

#### 4.2.1. Posterior Expected Number of Targets (PENT)

In 2003, Mahler [3] showed that the Csiszár information-theoretic objective function and geometric functionals lead to tractable sensor management algorithms when used in conjunction with the multi-hypothesis correlator (MHC) filtering algorithms. In 2004, Mahler et al. [5] proposed a “probabilistically natural” sensor management objective function called the posterior expected number of targets (PENT), constructed using an optimization strategy called “maxi-PIMS”. PENT was introduced for the control of sensors with a finite field-of-view (FoV), for the purpose of selecting the action that will maximize the posterior expected number of objects (cardinality) returned after updating the multi-object density. Hence, in the general sensor control framework shown in Figure 2, PENT is implemented by maximizing the reward function:

(43) $$R\left(u\right)=E_{{\tilde{\pi }}_{k}\left(\cdot ;u\right)}\left\{\left|{\tilde{X}}_{k}\right|\right\}=\int \left|{\tilde{X}}_{k}\right|{\tilde{\pi }}_{k}\left(X;u\right)\delta X.$$

Note that the term $E_{{\tilde{\pi }}_{k}\left(\cdot ;u\right)}\left\{\left|{\tilde{X}}_{k}\right|\right\}$ is indeed the Expected A Posteriori (EAP) estimate of posterior cardinality, and could be replaced with the Maximum A Posterior (MAP) estimate, turning the sensor control policy into the general form of:

(44) $$u^{*}=\underset{u\in U}{arg max}\left|{\hat{X}}_{k}\left(u\right)\right|.$$

Although PENT was originally introduced and its objective function was further formulated for PHD and MHC filters [3,5], the above general control policy is applicable with any multi-object filter in place, including the JPDA, MHT, MB, LMB and GLMB filters. Here are some examples:

When a PHD filter with SMC implementation is used as the multi-object system, the above control policy is given by:

(45) $$\begin{array}{ccc} u^{*} & = & \underset{u\in U}{arg max}\int_{X}\tilde{v_{k}}\left(x;u\right)dx \end{array}$$

(46) $$\begin{array}{ccc} & = & \sum_{i=1}^{N}{\tilde{w}}_{k,i}\left(u\right). \end{array}$$

Assuming that a multi-Bernoulli filter or an LMB filter with SMC is in place, we have:

(47) $$\begin{array}{ccc} u^{*} & = & \underset{u\in U}{arg max}\sum_{i=1}^{M}{\tilde{r}}_{k}^{\left(i\right)}\left(u\right),\\ \mathrm{or} & & \end{array}$$

(48) $$\begin{array}{ccc} u^{*} & = & \underset{u\in U}{arg max}\sum_{\ell \in L}{\tilde{r}}_{k}^{\left(\ell \right)}\left(u\right). \end{array}$$

#### 4.2.2. Cardinality Variance-Based Sensor Control

The PENT method is particularly useful when sensors have limited FoV. An alternative task-driven approach towards sensor control is to prioritize the accuracy of the resulting cardinality estimate rather than its mean. In this approach, the objective function is the following cost to be minimized:

(49) $$C\left(u\right)=\sigma_{{\tilde{\pi }}_{k}\left(\cdot ;u\right)}^{2}\left\{\left|{\tilde{X}}_{k}\right|\right\}=\sum_{m=1}^{M}{\left(m-E_{{\tilde{\pi }}_{k}\left(\cdot ;u\right)}\left\{\left|{\tilde{X}}_{k}\right|\right\}\right)}^{2}\mathrm{Pr}\left(\left|{\tilde{X}}_{k}\right|=m\right).$$

Gostar et al. [14,15] derived this cost function for the applications where a multi-Bernoulli filter is used for multi-object tracking, as follows:

(50) $$C\left(u\right)=\sum_{i=1}^{M}{\tilde{r}}_{k}^{\left(i\right)}\left(u\right)\left[1-{\tilde{r}}_{k}^{\left(i\right)}\left(u\right)\right].$$

Similarly, with an LMB filter, the cost function is given by:

(51) $$C\left(u\right)=\sum_{\ell \in L}{\tilde{r}}_{k}^{\left(\ell \right)}\left(u\right)\left[1-{\tilde{r}}_{k}^{\left(\ell \right)}\left(u\right)\right].$$

Hoang et al. [13] proposed to use the maximum a posteriori (MAP) estimate of cardinality for variance calculations, i.e., to the following cost function:

(52) $$C\left(u\right)=\sum_{m=1}^{M}{\left(m-{\hat{\left|X\right|}}_{\mathrm{MAP}}\right)}^{2}\mathrm{Pr}\left(\left|{\tilde{X}}_{k}\right|=m\right),$$

where

$${\hat{\left|X\right|}}_{\mathrm{MAP}}=\underset{m=1:M}{arg max}\mathrm{Pr}\left(\left|{\tilde{X}}_{k}\right|=m\right)$$

is the MAP estimate of cardinality. With an LMB filter, they showed the cost function is given by:

(53) $$C\left(u\right)=\sum_{i=1}^{M}{\tilde{r}}_{k}^{\left(i\right)}\left(u\right)\left(1-{\tilde{r}}_{k}^{\left(i\right)}\left(u\right)\right)+{\left[{\hat{\left|X\right|}}_{\mathrm{MAP}}-\sum_{i=1}^{M}{\tilde{r}}_{k}^{\left(i\right)}\left(u\right)\right]}^{2},$$

where the MAP cardinality estimate is calculated as follows:

(54) $${\hat{\left|X\right|}}_{\mathrm{MAP}}=\underset{m=1:M}{arg max}\left[\prod_{i=1}^{M}\left(1-{\tilde{r}}_{k}^{\left(i\right)}\left(u\right)\right)\sum_{1\leq i_{1}\neq \cdots \neq i_{m}\leq M}\left(\prod_{j=1}^{m}\frac{{\tilde{r}}_{k}^{\left(i_{j}\right)}\left(u\right)}{1-{\tilde{r}}_{k}^{\left(i_{j}\right)}\left(u\right)}\right)\right].$$

#### 4.2.3. Posterior Expected Error of Cardinality and States (PEECS)

In 2015, Gostar et al. [26] proposed a new cost function called Posterior Expected Error of Cardinality and States (PEECS) [26] in which a linear combination of the normalized errors of the number of objects and their estimated states is considered as the cost function for sensor control,

(55) $$C\left(u\right)=\eta \;\xi_{|X|}^{2}\left(u\right)+\left(1-\eta \right)\xi_{X}^{2}\left(u\right),$$

where $\xi_{|X|}^{2}\left(u\right)$ and $\xi_{X}^{2}\left(u\right)$ denote the normalized variances of the cardinality and state estimates, respectively, and η is a user-defined constant to determine emphasis on accuracy of cardinality estimates versus the state estimates. The normalized variance is given by (50) but divided by M/4, which is the maximum variance corresponding to the worst case where all probabilities of existence are 0.5,

(56) $$\xi_{|X|}^{2}\left(u\right)=\frac{4}{M}\sum_{i=1}^{M}{\tilde{r}}_{k}^{\left(i\right)}\left(u\right)\left[1-{\tilde{r}}_{k}^{\left(i\right)}\left(u\right)\right].$$

The normalized variance of state estimates depends on the application and the variables included in the single-object state. Consider an application where the principal interest is in localization error in a 2D space. Let us assume that the state vector includes the *x*- and *y*-coordinates of the object, denoted by $p_{x}$ and $p_{y}$, respectively. Consider a multi-Bernoulli filter being used as the MOT, and implemented using the SMC method in which the (i)-th Bernoulli component of the pseudo posterior ${\tilde{\pi }}_{k}\left(.;u\right)$ is parameterized by its probability of existence ${\tilde{r}}_{k}^{\left(i\right)}\left(u\right)$ and its density approximated by particles and weights ${\left\{\left({\tilde{w}}_{k,j}^{\left(i\right)}\left(u\right),x_{k,j}^{\left(i\right)}\right)\right\}}_{j=1}^{J^{\left(i\right)}}$ where the location components of the particle $x_{k,j}^{\left(i\right)}$ are denoted by $p_{x,k,j}^{\left(i\right)}$ and $p_{y,k,j}^{\left(i\right)}$. Then, the error variance associated with the (i)-th Bernoulli component is given by:

(57) $$\xi_{x^{\left(i\right)}}^{2}\propto \sigma_{p_{x}^{\left(i\right)}}^{2}\sigma_{p_{y}^{\left(i\right)}}^{2},$$

where $\sigma_{p_{x}^{\left(i\right)}}^{2}$ and $\sigma_{p_{y}^{\left(i\right)}}^{2}$ are the variances of *x*-coordinate and *y*-coordinate estimates of the possible object associated with the (i)-the Bernoulli component, and are given by:

(58) $$\begin{array}{ccc} \sigma_{p_{x}^{\left(i\right)}}^{2} & = & \sum_{j=1}^{J^{(}i)}{\tilde{w}}_{k,j}^{\left(i\right)}\left(u\right)\;{\left[p_{x,k,j}^{\left(i\right)}\right]}^{2}-{\left[\sum_{j=1}^{J^{(}i)}{\tilde{w}}_{k,j}^{\left(i\right)}\left(u\right)\;p_{x,k,j}^{\left(i\right)}\right]}^{2}, \end{array}$$

(59) $$\begin{array}{ccc} \sigma_{p_{y}^{\left(i\right)}}^{2} & = & \sum_{j=1}^{J^{(}i)}{\tilde{w}}_{k,j}^{\left(i\right)}\left(u\right)\;{\left[p_{y,k,j}^{\left(i\right)}\right]}^{2}-{\left[\sum_{j=1}^{J^{(}i)}{\tilde{w}}_{k,j}^{\left(i\right)}\left(u\right)\;p_{y,k,j}^{\left(i\right)}\right]}^{2}. \end{array}$$

The maximum of the above variances occur when all particle weights are equal to $\frac{1}{J^{\left(i\right)}}$, which leads to:

(60) $$\begin{array}{ccc} \max\left(\sigma_{p_{x}^{\left(i\right)}}^{2}\right) & = & \frac{1}{J^{\left(i\right)}}\left(1-\frac{1}{J^{\left(i\right)}}\right)\left(\sum_{j=1}^{J^{(}i)}{\left[p_{x,k,j}^{\left(i\right)}\right]}^{2}\right), \end{array}$$

(61) $$\begin{array}{ccc} \max\left(\sigma_{p_{y}^{\left(i\right)}}^{2}\right) & = & \frac{1}{J^{\left(i\right)}}\left(1-\frac{1}{J^{\left(i\right)}}\right)\left(\sum_{j=1}^{J^{(}i)}{\left[p_{y,k,j}^{\left(i\right)}\right]}^{2}\right) \end{array}$$

and the normalized state variance for the (i)-th Bernoulli component is calculated by:

(62) $$\xi_{x^{\left(i\right)}}^{2}=\frac{\sigma_{p_{x}^{\left(i\right)}}^{2}\;\sigma_{p_{y}^{\left(i\right)}}^{2}}{\max\left(\sigma_{p_{x}^{\left(i\right)}}^{2}\right)\;\max\left(\sigma_{p_{y}^{\left(i\right)}}^{2}\right)}.$$

The total state variance (which is still normalized) is given by:

(63) $$\xi_{X}^{2}=\frac{\sum_{i=1}^{M}{\tilde{r}}_{k}^{\left(i\right)}\left(u\right)\xi_{x^{\left(i\right)}}^{2}}{\sum_{i=1}^{M}{\tilde{r}}_{k}^{\left(i\right)}\left(u\right)}.$$

### 4.3. Selective Sensor Control

All the methods discussed so far deal with the sensor control problem for multi-target tracking of a group of targets or a target ensemble. The labeled random finite set filters such as LMB and GLMB filters allow for tracking the target trajectories along with estimating the number of targets and their states within the stochastic filtering scheme. Therefore, with such filters used, such as the MOT, one can control the sensor(s) towards acquiring most useful measurements for the purpose of tracking the *targets of interest* (ToIs), which are those with specific labels of interest.

#### 4.3.1. Maximum Confidence in Existence

Panicker et al. [16] investigated how the target label information returned by an LMB filter can be effectively used for sensor control focused on some ToIs. An intuitive solution is also proposed in [16] for scenarios in which one or more of the ToIs temporarily disappear from the tracking scene. Their method is a task-driven sensor control routine with a cost function that is dependent on the pseudo-updated states of only the ToIs.

This approach is based on maximizing the expected confidence of the filter’s inference on the existence of the ToIs. After the pseudo-update of the LMB density, the filter returns an estimate of the total number of existing ToIs, given by the cardinality mean $\sum_{\ell \in L_{\mathrm{ToI}}}{\tilde{r}}_{k}^{\left(\ell \right)}\left(u\right),$ where $L_{\mathrm{ToI}}$ is the set of labels of interest and ${\tilde{r}}_{k}^{\left(\ell \right)}\left(u\right)$ is the pseudo-updated probability of existence for the object with label *ℓ*. The confidence in this estimate is inversely related to the variance of cardinality. Therefore, to maximize the confidence, the following cost function is minimized:

(64) $$C\left(u\right)=\sum_{\ell \in L_{\mathrm{ToI}}}{\tilde{r}}_{k}^{\left(\ell \right)}\left(u\right)\left(1-{\tilde{r}}_{k}^{\left(\ell \right)}\left(u\right)\right).$$

#### 4.3.2. Selective-PEECS

As an alternative selective sensor control solution, the Selective-PEECS approach [16] employs the PEECS cost function [26]. The selective-PEECS cost function is similar to PEECS in Equation (55) with the difference that the normalized variances of cardinality and state terms are computed by summing over the labels of interest. More specifically, we have:

(65) $$\begin{array}{ccc} \xi_{|X|}^{2}\left(u;L_{\mathrm{ToI}}\right) & = & \frac{4}{|L_{\mathrm{ToI}}|}\sum_{\ell \in L_{\mathrm{ToI}}}{\tilde{r}}_{k}^{\left(\ell \right)}\left(u\right)\left[1-{\tilde{r}}_{k}^{\left(\ell \right)}\left(u\right)\right], \end{array}$$

(66) $$\begin{array}{ccc} \xi_{X}^{2} & = & \sum_{\ell \in L_{\mathrm{ToI}}}{\tilde{r}}_{k}^{\left(\ell \right)}\left(u\right)\xi_{x^{\left(\ell \right)}}^{2}/\sum_{\ell \in L_{\mathrm{ToI}}}{\tilde{r}}_{k}^{\left(\ell \right)}\left(u\right). \end{array}$$

### 4.4. Extension to Multi-Sensor Control

**Figure 4:**
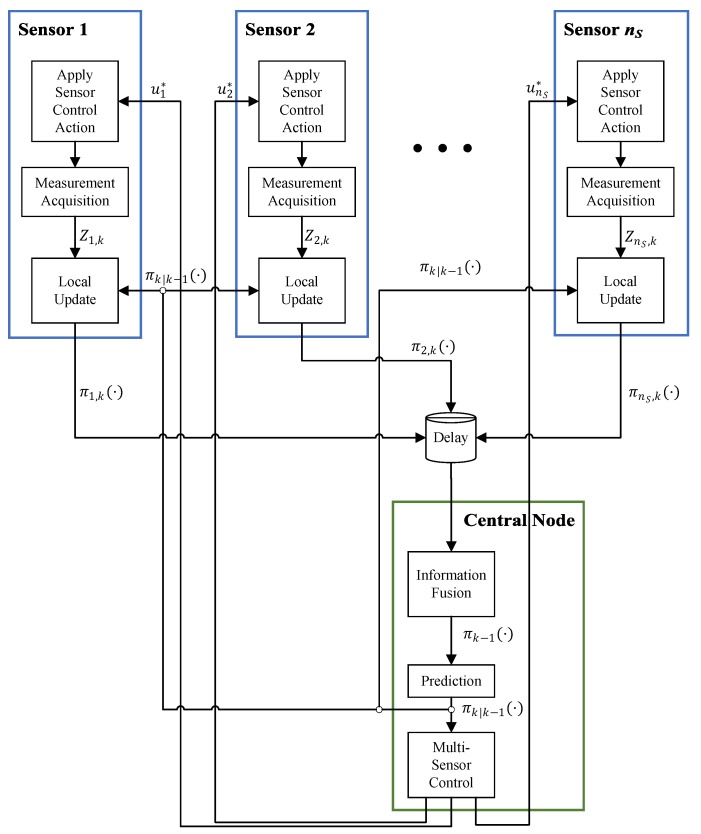
The general block diagram of a multi-sensor control system within a centralized sensor network.

Most of the work discussed earlier uses a single sensor control strategy for tracking multiple targets. In a multi-sensor scenario, the control solution is straightforward if the sensors form a centralized network. Consider $n_{S}$ sensor nodes that are all connected to a central node as shown in Figure 4. At each time *k*, in each node $S_{i}$, the sensor receives a command $u_{i}\in U$ from the central node and changes its state (e.g., moves, spins, changes its gain and so on) accordingly. Then, it generates a measurement set of detections $Z_{i}$ that is locally used to run the update step of a multi-object filter. All of the local posteriors, $\pi_{1,k},\pi_{2,k},\ldots ,\pi_{n_{S},k}$ are then communicated back to the central node.

In the central node, the received local posteriors are fused and the resulting multi-object density is used to run the prediction step of the multi-object filter centrally. The predicted prior is then used for two purposes: (i) it is communicated back to each sensor node for the local update to run in the next time, and (ii) it is input to a *multi-sensor control* routine that generates an $n_{S}$-tuple multi-sensor control command $\left(u_{1}^{*},u_{2}^{*},\ldots ,u_{n_{S}}^{*}\right)\in U^{n_{S}}$ and sends each control command to the corresponding sensor node.

Algorithm 1 shows the most straightforward process that can be implemented for the “Multi-Sensor Control” block in Figure 4. In the first step, a multi-object estimate ${\hat{X}}_{k|k-1}$ is computed from the prior. It is then used to calculate PIMS for each sensor node after being hypothetically actuated based on each possible control command. The PIMS is then used to run a pseudo-update step in the central node, which results in a pseudo-posterior for each node and each control command.

After all the possible pseudo-posteriors are computed for all sensor nodes and all control commands, for each $n_{S}$-tuple multi-sensor control command $\left(u_{1},u_{2},\ldots ,u_{n_{S}}\right)\in U^{n_{S}},$ all the corresponding pseudo-posteriors at different sensor nodes are fused, and the resulting pseudo-posterior is used to compute the objective function. In Algorithm 1, it is a reward function that is maximized to return the optimal multi-sensor control command $\left(u_{1}^{*},u_{2}^{*},\ldots ,u_{n_{S}}^{*}\right)$.

**Note:** The reward (or cost) function used in line 11 of Algorithm 1 can be any of the functions discussed in this section, divergence-based or task-driven or selective.

It is also important to note that the information fusion operation used in line 10 of Algorithm 1 must be the same operation that is used for fusion of real posteriors in the central node (the “Information Fusion” block in Figure 4). One of the widely used methods for fusion of multiple posteriors is the Generalized Covariance Intersection (GCI) rule that is employed for consensus-based fusion of multiple multi-object densities of various forms. It has been used for fusion of Poisson multi-object posteriors of multiple local PHD filters [48], multi-Bernoulli densities of local multi-Bernoulli filters [49], i.d.d. clusters densities of several CPHD filters [50] and GLMB densities of several local Vo–Vo filters or LMB densities of several LMB filters [51].

**Algorithm 1** Step-by-step operations that run inside the multi-sensor control block in Figure 4.
1:**function**Multi_Sensor_Control($\pi_{k|k-1}\left(\cdot \right)$)2:    ${\hat{X}}_{k|k-1}\leftarrow$Estimate($\pi_{k|k-1}\left(\cdot \right)$)3:    **for** $i=1:n_{S}$ **do**4:        **for** $u_{i}\in U$ **do**5:           $\tilde{Z}\left(u_{i}\right)\leftarrow$PIMS(${\hat{X}}_{k|k-1};u_{i},i$)6:           ${\tilde{\pi }}_{i,k}\left(\cdot ;u_{i}\right)\leftarrow$Update($\pi_{k|k-1}\left(\cdot \right),\tilde{Z}\left(u_{i}\right)$)7:        **end for**8:    **end for**9:    **for** $\left(u_{1},\ldots ,u_{n_{S}}\right)\in U^{n_{S}}$ **do**10:        ${\tilde{\pi }}_{k}\left(\cdot ;u_{1},\ldots ,u_{n_{S}}\right)\leftarrow$Fuse(${\tilde{\pi }}_{1,k}\left(\cdot ;u_{1}\right),\ldots ,{\tilde{\pi }}_{n_{S},k}\left(\cdot ;u_{n_{S}}\right)$)11:        $R(u_{1},\ldots ,u_{n_{S}})\leftarrow$Reward(${\tilde{\pi }}_{k}\left(\cdot ;u_{1},\ldots ,u_{n_{S}}\right)$)12:    **end for**13:    $\left(u_{1}^{*},\ldots ,u_{n_{S}}^{*}\right)\leftarrow {arg max}_{\left(u_{1},\ldots ,u_{n_{S}}\right)\in U^{n_{S}}}R\left(u_{1},\ldots ,u_{n_{S}}\right)$14:    **return** $\left(u_{1}^{*},\ldots ,u_{n_{S}}^{*}\right)$15:
**end function**



#### ARAPP (Accelerated Ratio of Absence to Presence Probability) Method

As a multi-sensor extension to the selective sensor control solution discussed earlier, the ARAPP approach [52] employs the RAPP (Ratio of Absence to Presence Probability) cost function [52]. This method optimizes a closed-form objective function called RAPP which can be calculated directly after the prediction step in the central node of the sensor network. The new cost function is computed before the pseudo-update operations, and hence a large amount of computation is saved. The simulation results in the paper suggest that the proposed methods lead to significant improvements in terms of tracking accuracy of objects of interest, compared to using the non-selective sensor control methods. Numerical experiments indicate that the proposed method significantly outperforms the common non-selective sensor control methods. When compared to selective-PEECS, the state-of-the-art method for selective sensor control, it is seen to perform similarly in terms of the mean-square-error (MSE) of tracking of the targets of interest, but is significantly (eight times) faster than the selective-PEECS method, which suggests a substantial reduction in the computational overhead.

## 5. Comparative Simulation Results

This section presents a few samples of recent works in which the performance of various sensor control algorithms are compared through simulations that involve multiple manoeuvring targets. In all the simulations, the single target state includes the location coordinates and speeds of the target, i.e., $x={\left[\begin{array}{cccc} p_{x} & {\dot{p}}_{x} & p_{y} & {\dot{p}}_{y} \end{array}\right]}^{⊤}.$ Each target randomly moves from time k−1 to *k* according to $x_{k}=Fx_{k-1}+e_{k}$ where $F=\mathrm{diag}(F_{2},F_{2},1)$ and $e_{k}∼N\left(0,Q\right)$ and we have:

$$F_{2}=\left[\begin{array}{cc} 1 & T\\ 0 & 1 \end{array}\right],\;\;Q=\sigma_{x}^{2}\;\mathrm{diag}\left(Q_{2},Q_{2},1\right),\;\;Q_{2}=\left[\begin{array}{cc} T^{4}/4 & T^{3}/2\\ T^{3}/2 & T^{2} \end{array}\right]$$

in which *T* is the sampling time, and $\sigma_{x}^{2}$ is the noise power.

A controllable sensor is used to detect the targets and return their range and bearing angles in a surveillance area. The probability of the sensor detecting an object depends on the distance between the sensor and the object,

(67) $$p_{D}\left(s,p\right)=\left\{\begin{array}{cc} 0.99, & \mathrm{if}\;||s-p||\leq Ro,\\ \max\left(0,0.99-h\left(||s-p||-R_{0}\right)\right), & \mathrm{otherwise}, \end{array}\right.$$

where $p={\left[p_{x}\;p_{y}\right]}^{⊤}$ and $s={\left[s_{x}\;s_{y}\right]}^{⊤}$ are the object and sensor locations, respectively. Conditional on detection, the measurement returned by the sensor is noisy, formulated by:

(68) $$z=\left[\begin{array}{c} ||s-p||+n_{\rho }\\ ∡\left(s-p\right)+n_{\theta } \end{array}\right],$$

where $n_{\rho }$ and $n_{\theta }$ are range and bearing measurement noise, and both normally distributed with zero mean but different and varying variances,

(69) $$\begin{array}{ccc} n_{\rho } & ∼ & N\left(0,\sigma_{\rho }^{2}\right), \end{array}$$

(70) $$\begin{array}{ccc} n_{\theta } & ∼ & N\left(0,\sigma_{\theta }^{2}\right). \end{array}$$

Similar to the probability of detection, the noise power terms are also dependent on the sensor-object distance:

(71) $$\begin{array}{ccc} \sigma_{\rho } & = & \sigma_{\rho ,0}+\beta_{\rho }||s-{p||}^{2}, \end{array}$$

(72) $$\begin{array}{ccc} \sigma_{\theta } & = & \sigma_{\theta ,0}+\beta_{\theta }||s-{p||}^{2}. \end{array}$$

**Figure 5:**
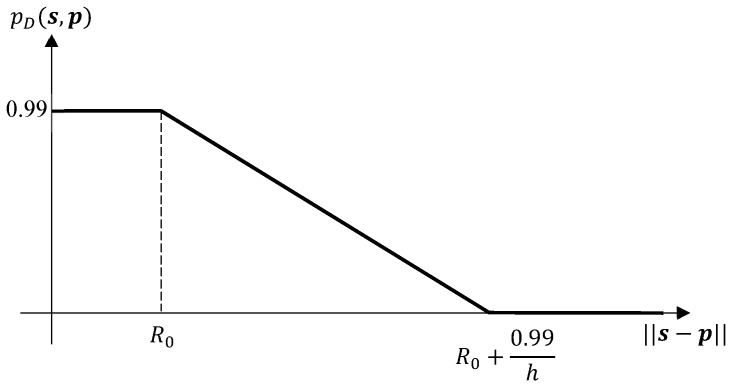
The detection probability given by (67) decreases as the sensor-object distance increases.

The target position is a function of the pulse transit time between the sensor and target and is proportional to the distance between them. In addition, the delay of received echo is proportional to the distance to target. In a recent work, Yong et al. [53] state that, although the radar system errors are impacted by various factors such as the altitude of the sensor (radar), range and elevation between the sensor and target, the main factors of influence are the range and elevation. Importantly, the probability of detection decreases with the sensor-object distance (see Figure 5), and the power of measurement noise increases with the sensor-object distance. Hence, in this case, the sensor is generally expected to return more accurate measurements (that include less noise and misdetections) and the sensor control solution is expected to drive the sensor towards the centre of all objects as they manoeuvre.

The above scenario and models for target motion and measurement uncertainties is one of the most common scenarios considered for performance evaluation in the sensor control literature. Table 2 shows a list of such methods and the scenarios considered in them.

**Figure 6:**
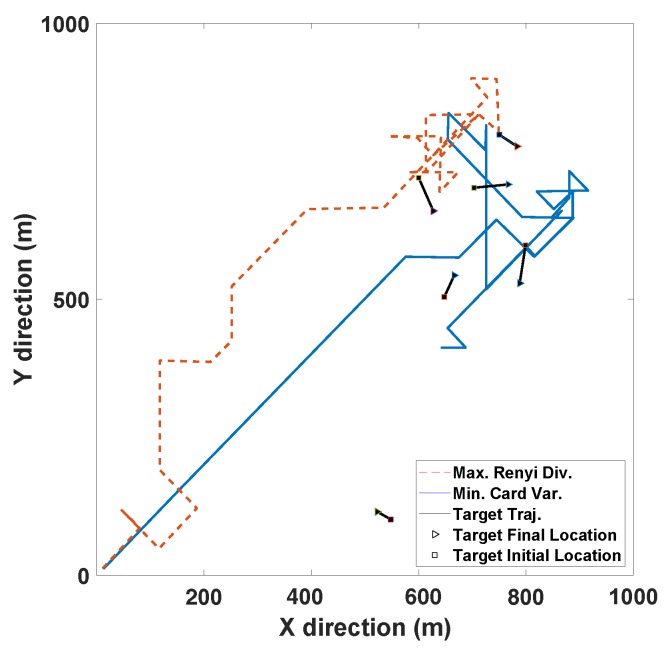
Sensor trajectories of the MAP cardinality variance-based and Rényi divergence-based sensor control methods.

Figure 6 illustrates the typical resultant sensor trajectories for Rényi divergence-based and the MAP cardinality variance-based sensor control strategies as reported by Hoang et al. [32]. In their simulation, five targets manoeuvre in a 1000 m×1000 m surveillance area. The sensor starts from the origin, and at each time *k*, its previous location $s_{k-1}={\left[s_{x,k-1}\;s_{y,k-1}\right]}^{⊤}$ varies to one of the admissible locations in the following control command space:

$$s_{k}\in U_{k}=\left\{{\left[\begin{array}{cc} s_{x,k-1}+i_{R}\;\Delta R\cos\left(\frac{2\pi \;i_{\theta }}{N_{\theta }}\right)\;\; & \;\;s_{y,k-1}+i_{R}\;\Delta R\sin\left(\frac{2\pi \;i_{\theta }}{N_{\theta }}\right) \end{array}\right]}^{⊤}:\left(i_{R},i_{\theta }\right)\in \left[0:N_{R}\right]\times \left[0:N_{\theta }\right]\right\}.$$

**Figure 7:**
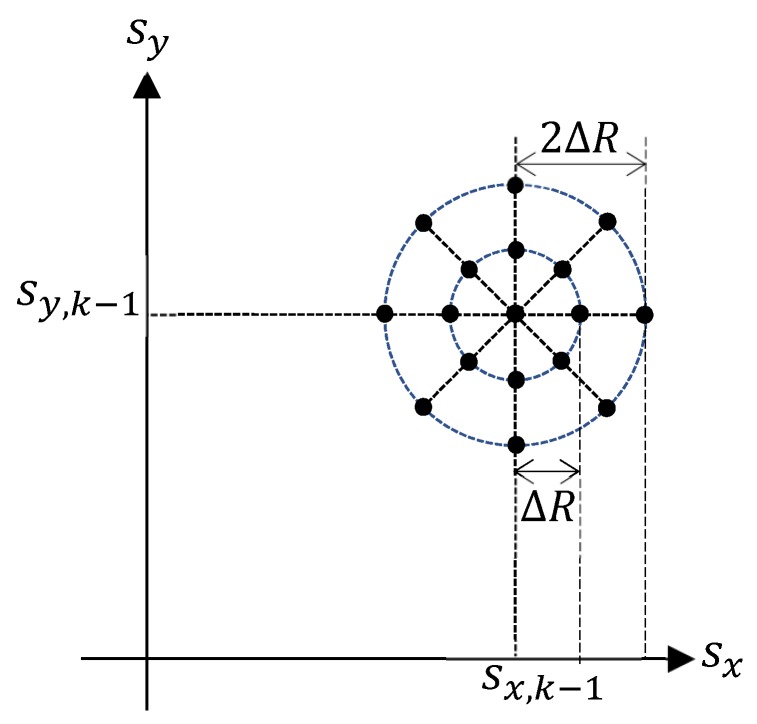
All the admissible movements of a sensor with parameters $N_{R}=2$ and $N_{\theta }=8$ are shown as solid black circles.

In the experiment shown in Figure 6, we have borrowed the parameters ΔR=50m, $N_{R}=2$ and $N_{\theta }=8$. All the admissible sensor movements from the location $s_{k-1}={\left[s_{x,k-1}\;s_{y,k-1}\right]}^{⊤}$ are shown as solid black circles in Figure 7. The parameters of the detection profile in Equation (67) and measurement noise are chosen as:

$$\begin{array}{cccccc} R_{0} & = & 300\;m, & h & = & 5\times 10^{-4}\;m^{-1},\\ \sigma_{\rho ,0} & = & 1\;m, & \beta_{\rho } & = & 5\times 10^{-5}\;m^{-1},\\ \sigma_{\theta ,0} & = & \frac{\pi }{180}\;\mathrm{rad}, & \beta_{\theta } & = & 10^{-5}\;\mathrm{rad}\times m^{-1}. \end{array}$$

**Figure 8:**
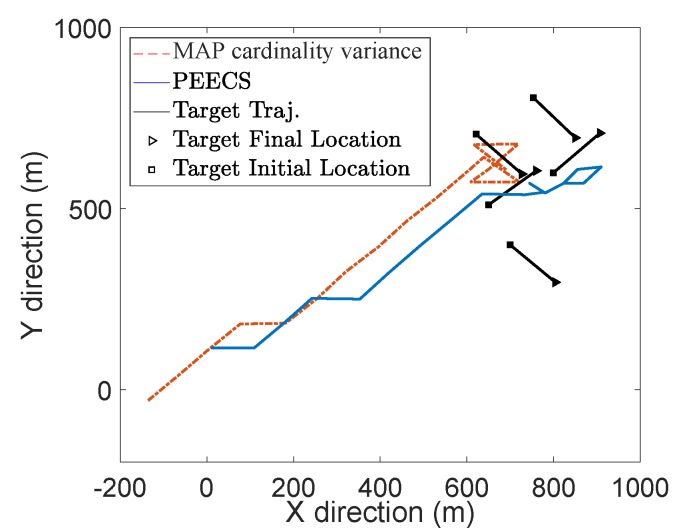
Sensor locations for PEECS [26] and Cardinality variance [32] cost functions (as reported in [26]).

**Figure 9:**
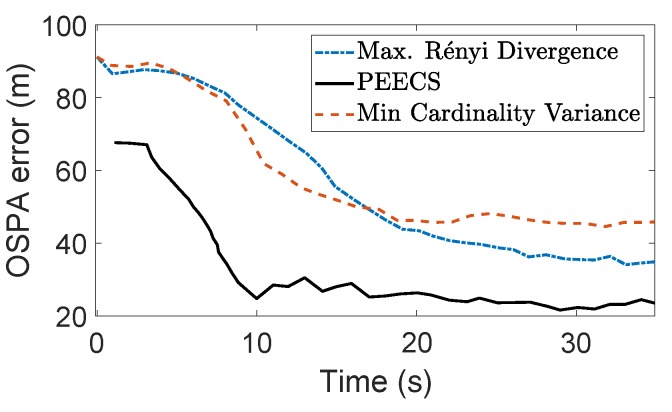
Optimal Sub-Pattern Assignment (OSPA) errors of PEECS compared to the sensor control methods suggested by Hoang et al. in [32] (as reported in [26]).

**Figure 10:**
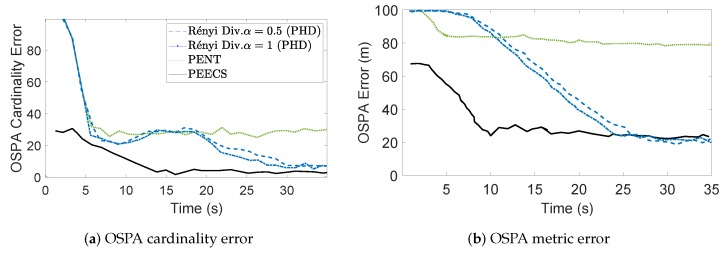
Estimation errors of the PEECS method compared to PHD-based sensor control methods (PENT [2] and Rényi divergence [12]) as reported in [26].

**Figure 11:**
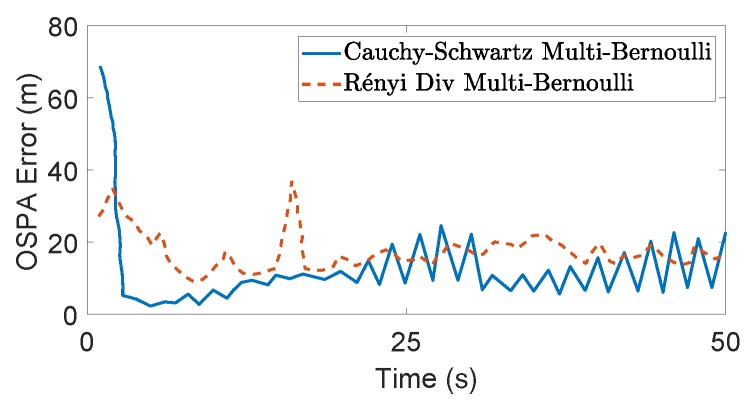
OSPA errors returned by the sensor control method based on Cauchy–Schwarz divergence [35] compared to the Rényi divergence-based sensor control [11], as reported in [35].

The task-driven PEECS method [26] is compared with the MAP cardinality variance method [32] and the resulting sensor locations are shown in Figure 8. Figure 9 compares the OSPA errors of PEECS and the Rényi divergence-based method suggested in [32]. As both cardinality and localization errors are considered in the cost function of PEECS, this method improves the sensor control performance for multi-target scenarios with high clutter. In Figure 10, the estimation errors of PEECS sensor control method are compared to the PHD-based methods [26]. Similarly, the OSPA errors returned by the sensor control method based on Cauchy–Schwarz divergence [35] are compared to Rényi divergence-based sensor control [11] as presented in Figure 11.

**Figure 12:**
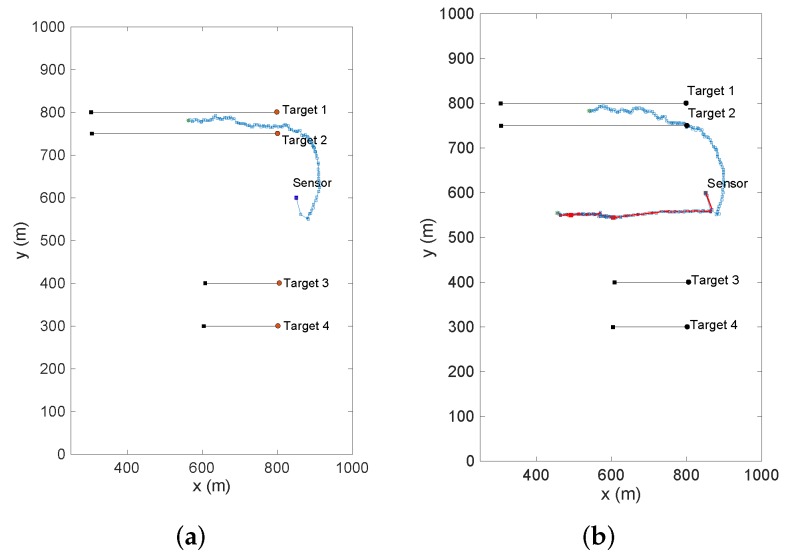
Sensor trajectories with non-selective (PEECS) and selective sensor control methods as reported in [16]: (**a**) comparison of the resulting sensor trajectories using PEECS [26] and selective-PEECS. [16], (**b**) sensor trajectory using maximum confidence method for sensor control solution [16].

**Figure 13:**
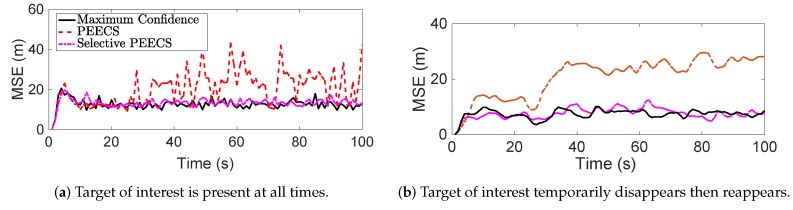
Mean square error of state estimates returned by selective sensor control methods (selective-PEECS and Maximum Confidence method) and non-selective PEECS as reported in [16].

Figure 12 and Figure 13 show the simulation results borrowed from [16] for a selective single-sensor multi-target scenario. While Figure 12a demonstrates the sensor path returned by the maximum confidence method, Figure 12b provides the sensor trajectories returned by the selective and non-selective PEECS sensor control methods. Comparing the trajectories, it is inferred that the selective sensor control methods definitely serve selective tracking applications better than the traditional non-selective methods. Figure 13a provides the mean squared error (MSE) returned on tracking the ToIs in order to compare the performance of selective method with PEECS [26], the non-selective sensor control routine. Figure 13b demonstrates the results obtained for the case of disappearing target. It is shown in [16] that tracking error for the ToIs is reduced and speed of computation is considerably increased, when selective sensor control is included.

## 6. Discussion on Performance Comparison

In this section, we provide a performance comparison for various methods considering the contributions made towards the sensor control. In the sensor control literature, performance enhancement is usually demonstrated in terms of reduction in tracking errors. The computational speed has not been a major focus for performance and comparison in the recent literature. Hence, this review covers the performance comparisons in terms of tracking accuracy in a comprehensive manner. However, acknowledging the importance of computation, we also provide a table for comparison of computational speed (Table 3), as presented in the recent literature on selective sensor control methods [52]. It compares the labelled RFS-based selective sensor control methods with a state-of-the-art non-selective sensor control method.

A comparative study of the Rényi divergence (information theoretic) and cardinality variance (task driven) approaches discussed by Hoang and Vo [32] imply that, although both the control methods perform well in cases with high target observability, in specific cases where the observability is low, their performance degrades. In such scenarios, Rényi divergence performs better than the other, but the Cardinality variance method results in a smoother sensor trajectory, as observed from Figure 6. On comparing the OSPA performance of these methods with PEECS sensor control, as discussed in [26] and observed from Figure 9, it is evident that PEECS exhibits better performance, primarily due to its structural emphasis on taking error terms into account that are similar to the terms that form the OSPA metric. PEECS performs similarly to the MAP cardinality variance cost function, differing in its use of variance of cardinality around the mean (statistical variance) instead of variance around the MAP estimate. In addition, errors in both the cardinality and state estimates impact the PEECS cost. Figure 10a,b indicate that the OSPA errors of PEECS converge to a minimum faster than PENT and PHD-based Rényi divergence methods.

Performance evaluation of these non-selective sensor control methods compared to selective control methods returns promising results for the tracking accuracy in terms of computational speed and performance metric (mean-squared error (MSE) of the state estimates of targets of interest). This is evident from Figure 13, which clearly shows significant improvements in state estimation errors for the ToIs with the selective methods such as Maximum confidence and selective-PEECS compared to the non-selective PEECS method.

Considering computational speed, due to lack of a generic closed-form solution, Rényi divergence-based sensor control is usually implemented with *set particle* approximations and hence is substantially more computationally expensive than task driven solutions such as PEECS whose cost functions are computationally effective. Indeed, PEECS sensor control is shown to be at least four times faster than the PHD-based sensor control methods [12]. The closed form solution for Cauchy–Schwarz divergence proposed by Gostar et al. [35] (in a multi-Bernoulli filtering scheme) seems to outperform the computational speed of the information-theoretic sensor control methods, while maintaining comparable tracking accuracy. In Figure 11, the OSPA errors returned by the sensor control method based on Cauchy–Schwarz divergence [35] is shown in comparison to the Rényi divergence-based sensor control [11], as reported in [35]. It can be inferred from Table 3 that, in a multi-sensor scenario, the ARAPP sensor control method is very efficient, making it 36 times faster than the non-selective PEECS, and eight times faster than the selective-PEECS method.

## 7. Conclusions and Future Directions

One of the most significant challenges with multi-sensor control is its computational cost due to the need for search in the multi-dimensional sensor command space $U^{n_{s}}$. Indeed, looking at lines 9–12 of Algorithm 1, for each $n_{s}$-tuple $\left(u_{1},\ldots ,u_{n_{s}}\right)\in U^{n_{s}}$, a fusion operation step followed by a reward calculation step need to be conducted. Algorithm 1 presents the most straightforward approach which involves an *excessive* search for the optimal multi-sensor control command. For practical feasibility, especially in real-time applications, there is a significant interest in alternative *accelerated* search routines.

Wang et al. [36] have recently introduced a guided search method to solve the multi-dimensional optimization problem inherent to multi-sensor control using an accelerated scheme inspired by the Coordinate Descent Method (CDM) [36]. This results in significant improvement in the runtime of the algorithm and also its real-time feasibility in the presence of multiple sensors. However, there is still a large room for improvement, as the CDM does not exploit the multi-object density and PIMS information at its core and is merely an accelerated search over the multi-dimensional space.

The computational cost can also be improved by devising new ways to calculate the objective function without the need to go through the pseudo-update steps of the filter, which can be very expensive. The main point of sensor control is due to the sensor’s detection profile being dependent on the sensor state. Therefore, this dependency might be formulated directly in a closed-form approximate derivation for the objective function, so that we can calculate it without the need to run the pseudo-update step for each admissible (multi-)sensor control command.

## Figures and Tables

**Table 1:** Sensor control categorization.

Reference	Sensor Control Method	Year	Task-Driven?	Information-Driven?	RFS-Based?
Mahler et al. [2]	Csiszár divergence	1998		✓	✓
Kreucher et al. [43]	Alpha divergence with JMPD	2003		✓	✓
Mahler et al. [5]	PENT for PHD and MHC filters	2004		✓	✓
Ristic et al. [11]	Rényi alpha divergence	2010		✓	✓
Ristic et al. [12]	Rényi divergence	2011		✓	✓
Hoang et al. [32]	a) Rényi divergence b) MAP estimate of cardinality variance	2014		✓	✓
Gostar et al. [15]	statistical mean of cardinality variance	2013	✓		✓
Gostar et al. [26]	PEECS	2015	✓		✓
Beard et al. [44]	Closed form solution of Cauchy-Schwarz (CS) divergence for GLMB densities	2015		✓	✓
Gostar et al. [34]	OSPA-based objective Function	2015	✓		✓
Gostar et al. [35]	Closed form solution of CS divergence for multi Bernoulli densities	2016		✓	✓
Jiang et al. [30]	CS divergence based JDM, IDM methods for Labeled RFS	2016		✓	✓
Beard et al. [33]	Void probability functional and CS divergence for GLMB filter	2017		✓	✓
Gostar et al. [45]	Void probability functional and CS divergence for LMB filter	2017		✓	✓
Wang et al. [36]	Multi sensor control with GCI and CDM for PEECS	2018	✓		✓
Panicker et al. [16]	Maximum confidence method and selective-PEECS	2018	✓		✓

**Table 2:** Sensor control scenarios in the recent sensor control and target tracking literature.

Sensor Control Solution	Tracking and Sensing Scenario Description
Ristic et al. [11]	Constant number of static targets (2 targets), probability of detection is homogeneous and constant across the area of surveillance, standard deviation of range measurements depends on the distance between the sensor and target. Range only controllable sensor, 17 control options.
Ristic et al. [12]	5 targets, pD depends on distance between sensor and object, Standard deviation of range measurements depends on the distance between sensor and target constant velocity target motion model. Range only controllable sensor, 17 control options.
Hoang et al. [13]	Maximum of 5 targets, pD depends on distance between sensor and object, constant velocity target motion model. Range only controllable sensor, 17 control options.
Gostar et al. [14]	5 targets, pD depends on distance between sensor and object, constant velocity target motion model. Range only controllable sensor, 17 control options.
Panicker et al. [16]	4 targets, 2 ToIs (Targets of Interest), pD depends on distance between sensor and object, Nearly constant velocity target motion model. Controllable mobile sensor, 9 control options, Selective sensor control.
Panicker et al. [52]	10 targets, 2 ToIs, Coordinated Turn (CT) target motion model, pD depends on distance between sensor and object. 4 sensors, 11 possible sensor control commands.
Hoang et al. [32]	5 moving targets, constant velocity motion model, pD is range dependent. Single mobile range and bearing sensor, Range-dependent sensor noise.
Beard et al. [33]	8 targets, Discrete white noise acceleration motion model, pD is range dependent. Single mobile range and bearing sensor, measurement noise on the bearings is constant for all targets, but the range noise is state-dependent, increasing as the true range between the sensor and target increases.
Gostar et al. [26]	5 targets, pD range dependent, Case study 1: Pseudo stationery targets, Case study 2: Nearly constant turn model. Single mobile sensor, Range-dependent sensor noise, Case study 1: Controllable range only sensor, Case study 2: Controllable range and bearing sensor
Gostar et al. [34]	15 pseudo stationery targets, pD distance dependent. Bearing and range mobile sensor.
Gostar et al. [35]	10 pseudo stationery targets, pD distance dependent. Single sensor, 17 control commands.
Kreucher et al. [43]	3 targets, white noise acceleration target motion model. Single bearings-only sensor, constant velocity sensor motion model.
Gostar et al. [45]	6 targets, coordinated turn model, pD range dependant. Single range and bearing mobile sensor.

**Table 3:** Comparison of computational speed achieved by recent sensor control methods as reported in [52].

Sensor Control Method	Type	Execution Time per MC Run
PEECS	Non-selective	$2.280\times 10^{4}$ s
Selective-PEECS	Selective	$4.975\times 10^{3}$ s
ARAPP	Selective	$6.200\times 10^{2}$ s

## Abbreviations

The following abbreviations are used in this manuscript:

POMDP	Partially Observable Markov Decision Process
SMC	Sequential Monte Carlo
RFS	Random Finite Set
LMB	Labeled Multi-Bernoulli
GLMB	Generalized Labeled Multi-Bernoulli
MAP	Maximum A Posteriori
MC	Monte Carlo
CS	Cauchy–Schwarz
PHD	Probability Hypothesis Density
MHC	Multi-Hypothesis Correlator
JMPD	Joint Multitarget Probability Densities
PENT	Posterior Expected Number of Targets
PEECS	Posterior Expected Error of Cardinality and States
RAPP	Ratio of Absence to Presence Probability
ARAPP	Accelerated RAPP
GCI	Generalized Covariance Intersection
CDM	Coordinate Descent Method
ToI	Target of Interest
